# Vivax malaria in an Amazonian child with dilated cardiomyopathy

**DOI:** 10.1186/1475-2875-13-61

**Published:** 2014-02-18

**Authors:** Antonio C Martins, Jamille B Lins, Luana MN Santos, Licia N Fernandes, Rosely S Malafronte, Teresa C Maia, Melissa CV Ribera, Ricardo B Ribera, Monica da Silva-Nunes

**Affiliations:** 1 Health Sciences Centre, Federal University of Acre, BR 364 KM 04, Rio Branco, Acre, Brazil; 2 Pediatrics, Children’s Hospital, Rio Branco, Acre, Brazil; 3 Tropical Medicine Institute, University of São Paulo, São Paulo, Brazil

**Keywords:** Vivax malaria, Amazon, Dilated cardiomyopathy, Cardiac dysfunction, Cytomegalovirus infection

## Abstract

A child living in the Brazilian Amazon region who had had vivax malaria at the age of 11 months was admitted three months later with a history of progressive dyspnoea and fever, which culminated in respiratory distress and severe dilated cardiomyopathy at hospital admission in a malaria-free area. She received treatment for cardiac insufficiency and was tested for malaria with two thick blood smears, which were negative. There was general improvement of cardiorespiratory function in the next two weeks, but in the third week of hospital admission, there was re-appearance of fever, severe anaemia, severe plaquetopaenia, and respiratory distress. A third thick blood smear was positive for *Plasmodium vivax* mono-infection, which was confirmed by molecular methods. A serological panel with the most prevalent infectious agents known to cause myocarditis was performed, and specific anti-cytomegalovirus (CMV) IgM and elevated levels of anti-CMV IgG were also detected in the serum. After treatment for malaria, there was improvement of respiratory distress, although cardiac function did not recover. She was discharged home with drugs for cardiac insufficiency and is currently under follow-up with a paediatric cardiologist as an outpatient. This report presents a young child with several episodes of vivax malaria who suffers from cardiac insufficiency, probably related to CMV-induced myocarditis.

## Background

Vivax malaria is an important disease responsible for up to 400 million infections in endemic areas of Asia, Latin America, and some parts of Africa. In recent years, it has been shown that this so-called benign malaria can result in severe presentation with multiple organ dysfunction and life-threatening disease. The majority of severe vivax malaria cases have been reported in India [1-4], but many cases occurred in other countries where there is active transmission, such as Ethiopia [5], Sudan [6], Cambodia [7], Colombia [8], and Brazil [9-12]. Criteria for diagnosing severe vivax malaria, as opposed to severe falciparum malaria, are still not clearly defined, since some biological features of these two parasites are different, and in the meantime the WHO definition for severe falciparum malaria is used [13].

Vivax malaria is an important morbidity in children living in countries with endemic transmission. At the same time, children are also susceptible to other infections, such as viral diseases, because they still lack specific immunity to most of the pathogens in their first years of life, and co-morbidities can occur, especially in underdeveloped and developing regions, such as the Amazon.

This is the report of a case of acute myocarditis and dilated cardiomyopathy associated with cytomegalovirus (CMV) infection and vivax malaria in an infant residing in the Brazilian Amazon region.

## Case report

A 13-month old girl living in the rural area of Cruzeiro do Sul, in Acre state (a small town of 73,000 inhabitants that notified 16,500 malaria cases in 2012) was admitted to a local hospital facility on September 9^th^, 2013 with a complaint of shortness of breath for approximately 30 days prior to the admission. Her parents referred to productive cough and fever in the preceding days. Her medical history (referred by parents) included a three-day hospital admission at the age of 11 months (June 2013) for respiratory symptoms, which were considered to be ‘pneumonia', according to parents report. Since she had fever and lived in an area of malaria transmission, she was tested for malaria. Malaria records showed that she was diagnosed with vivax malaria, confirmed by microscopy. Malaria treatment was initiated with oral chloroquine (25 mg/kg over three days) and primaquine (0.5 mg/kg/day over seven days) while she was in the hospital, and primaquine treatment was completed later at home, according to parents’ report. She had been born full-term, 3,695 kg in weight and 50 cm in length, from caesarean surgery. Her mother denied attending prenatal care during pregnancy.

**Figure 1:**
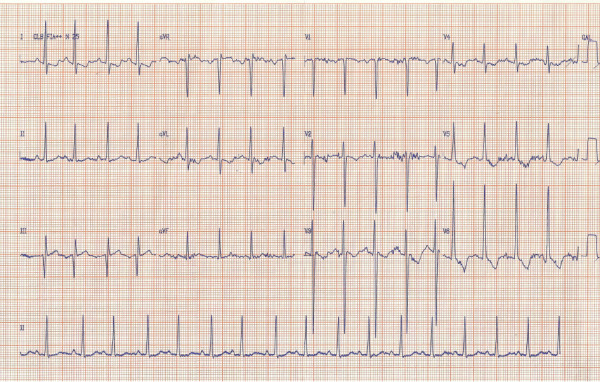
**Electrocardiogram in a child with dilated cardiomyopathy.** The electrocardiogram shows left ventricular and atrial overload.

**Figure 2:**
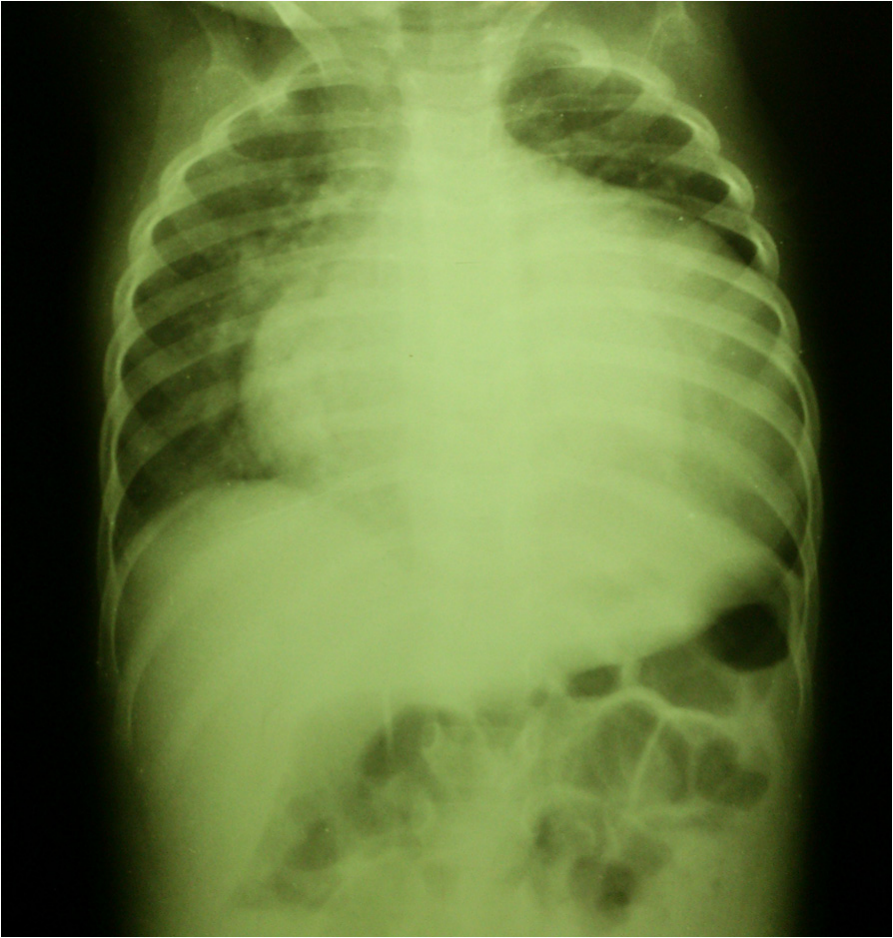
**Chest radiography showing cardiomegaly.** There is marked dilation of the left ventricle and increased pulmonary blood flow.

At hospital admission, she had signs of respiratory distress on physical examination, with wheezing, crackles, and nasal flare. Heart rate was 129 bpm, respiratory rate was 26 ipm. She measured 72 cm in length and her weight was 9 kg. There was mild hepatomegaly. During the next three days, her heart rate escalated to 162 bpm, respiratory frequency varied between 26 and 50 bpm, and axillary temperature ranged from 37 to 37.8°C. Based on signs of respiratory distress and productive cough, there was an initial diagnosis of pneumonia and she was started on ceftriaxone on the day of admission. The electrocardiogram showed sinus rhythm, left ventricular overload and left atrial overload (Figure 1). Chest X-ray showed pulmonary congestion and enlarged cardiac area (Figure 2). A thick smear for malaria diagnosis was negative on September 11^th^. According to the protocol stipulated by the Ministry of Health, 100 high power fields were examined before a negative diagnosis was issued. Unfortunately, rapid diagnostic tests for malaria were not available at the hospital at this time.

Because of her critical conditions, she was referred to a larger hospital in another city (Rio Branco) on September 13^rd^, with a diagnosis of cardiac failure of unknown origin, where she was seen by a paediatric cardiologist. On examination, she had severe dyspnoea (50 ipm) and a regular tachycardic heartbeat (140 bpm), with hypophonetic heart sounds. The first echocardiogram, performed on September 14^th^, showed dilated cardiomyopathy with severe systolic dysfunction: mildly dilated left atrium (27.2 mm), severely dilated left ventricle (60 × 50 mm), ejection fraction of 35%, severe mitral insufficiency, mild aortic valve insufficiency, and no signs of cardiac malformation. Laboratory examinations showed normal erythrocyte sedimentation rate, negative C-reactive protein test, normal CPK (26 U/l, range 26–155 U/l), increased value of CK-mB (42 U/l, normal: less than 25 U/L), AST 42 U/l, ALT 21 U/l. A haemogram revealed anaemia (haemoglobin 9.69 g/dL) and mild leukocytosis (10,900 cells/mm^3^), with 57% of granulocytes and 46% of lymphocytes, with normal platelet count (370,000/mm^3^). Another blood thick smear for malaria was performed on September 13^rd^, using the same criteria described before, which was also considered to be negative. She continued to receive ceftriaxone, and she was started on carvedilol, digoxin, furosemide, captopril, spironolactone, and oxacilin.

Between September 13^rd^ and 17^th^ she remained in stable condition with gradual decrease of heart rate (from 130 to 100 bpm) and respiratory rate (from 50 to 28 ipm). There was also disappearance of other clinical signs of respiratory distress.

On September 17^th^, she had diarrhoea with liquid stools which lasted nine days, with up to seven evacuations per day, accompanied by low-grade fever (37.2-37.6°C). On 19 September, the CKmB levels returned to normal (19.20 U/l). Ceftriaxone was terminated on September 20^th^ but the patient continued to present low-grade fever in the next days.

**Figure 3:**
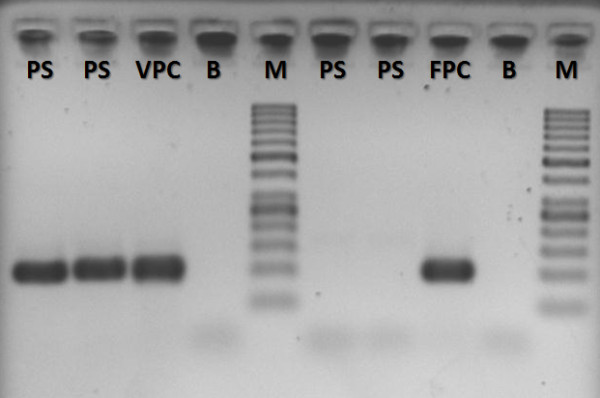
**Molecular diagnosis of *Plasmodium vivax *infection.** A DNA sample of the patient was submitted to nested PCR and it was positive for *P. vivax* mono-infection. PS = Patient sample; VPC = *P. vivax* positive control; FPC = *P. falciparum* positive control; B = Blank; M = Molecular weight marker.

**Figure 4:**
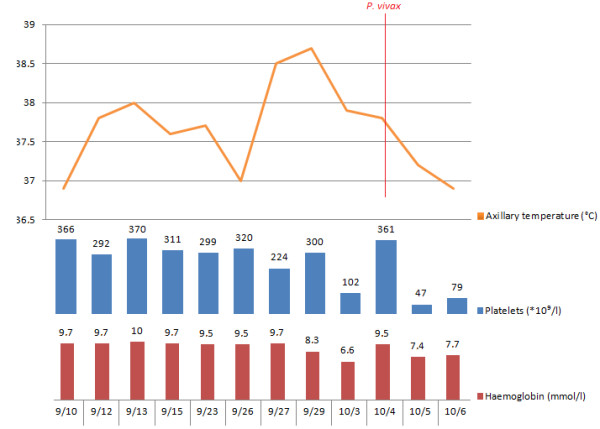
**Clinical evolution of fever, platelet counts and haemoglobin and its relation with malaria.** On admission (10/9) haemogobin level was 9.7 mmol/l and platelet count was normal. After a few days of admission axillary temperature increased, peaking on 29/9, when hourly antipyretics were prescribed. Four days later there was an acute drop on haemoglobin levels and platelet counts. The patient received a blood transfusion on 3/10, resulting in increased platelet count and haemoglobin levels. However, they declined again on 5/10. A positive smear for *P. vivax* was obtained in 4/10.

Ceftazidime and vancomycin were started on September 23^rd^, because fever did not cease and clinical conditions were getting worse day by day even after a full course of Ceftriaxone, making the clinicians very worrisome about her clinical status Diarrhoea ceased after three days of vancomycin, but axillary temperature continued to raise and reached 38.5°C despite antibiotic administration. On September 29^th^, she was started on dipirone every six hours; haemoglobin values were 8.3 g/dL. OnOctober 3^rd^ haemoglobin values had decreased to 6.6 g/dL, when she received a blood transfusion because of the acute drop in haemoglobin levels. There was worsening of dyspnoea (from 30 to 42 ipm) and tachycardia (from 110 to 124 bpm), and on October 4^th^, another thick blood smear for malaria was performed, which was positive for *P. vivax*, with approximately 10,000 parasites/mm^3^. Molecular diagnosis of *Plasmodium* species was performed using nested polymerase chain reaction [14,15], and it confirmed a mono-infection by *P. vivax* (Figure 3). Glucose levels were 74 mg/dL at this point. Standardized treatment with chloroquine (25 mg/kg over three days) and primaquine (0.5 mg/kg/day over seven days) according to Brazilian Ministry of Health [16] was started. After transfusion, haemoglobin levels reached 9.5 g/dL. However, a day later haemoglobin had decreased again to 7.7 g/dL and platelet count was 47,000/mm^3^. The patient was clinically anicteric, but no laboratory measurement of bilirubin was performed. Table 1 shows the result of haemograms and Figure 4 shows the relationship between fever, haemoglobin levels and platelet counts. After malaria diagnosis, the patient started to receive hourly antipyretics.

**Table 1:** Haemoglobin levels, leukocyte and differential counts, and platelet counts between initial admission on 10 September and malaria treatment in early October, 2013

	**Haemoglobin (mmol/l)**	**Leukocytes (mm**^**3**^**)**	**Segmented neutrophils %**	**Lymphocytes %**	Platelets (*10^**9**^**/l)**	**Important clinical events**
**10/9**	9.7	13,800	32	57	366	Dyspnoea, starts Ceftriaxone
**12/9**	9.7	7,900	45	49	292	
**13/9**	9.69	10,900	57	41	388	
**13/9**	10	14,900	48	46	370	
**15/9**	9.7	10,600	31	54	311	
**23/9**	9.5	32,200	70	16	299	Diarrhoea since 17/9; starts Ceftazidime + Vancomycin
**26/9**	9.5	9,500	53	36	320	
**27/9**	9.7	8,400	42	46	224	Fever 38.5°C
**29/9**	8.3	9,200	56	38	300	
**3/10**	6.6	12,100	53	13	102	Blood transfusion after haemogram
**4/10**	9.5	9,600	49	38	361	Smear positive for *P. vivax*
**5/10**	7.4	7,400	33	48	47	
**6/10**	7.7	14,800	32	48	79	

Reference values for 6 months to 2 years old children: 10.5 to 13.5 g/dL haemoglobin, 6,000– 17,500 leucocytes/μL, 15-35% segmented neutrophils, 45 – 6775% lymphocytes [17].

A panel of serologic tests for the most common infectious agents known to cause myocarditis in children was performed. Anti-CMV antibodies were detected on October 4^th^ (IgM of 2.66 for a cut-off > = 1.0 and IgG higher than 250, cut-off > 6.0), and six days later, IgM titres increased to 9.34 (cut-off > = 1.0). Laboratory investigation for other infectious agents (hepatitis B, hepatitis C, HIV, *Epstein Barr* virus, *Toxoplasma gondii, Trypanosoma cruzi*) were negative on two separate samples (October 4^th^ and October 10^th^). It was decided not to use gancyclovir because there was no evidence of possible clinical benefit at this point and since this drug can cause bone marrow toxicity, it may have exacerbated the anaemia and thrombocytopaenia if it had been used [18].

**Figure 5:**
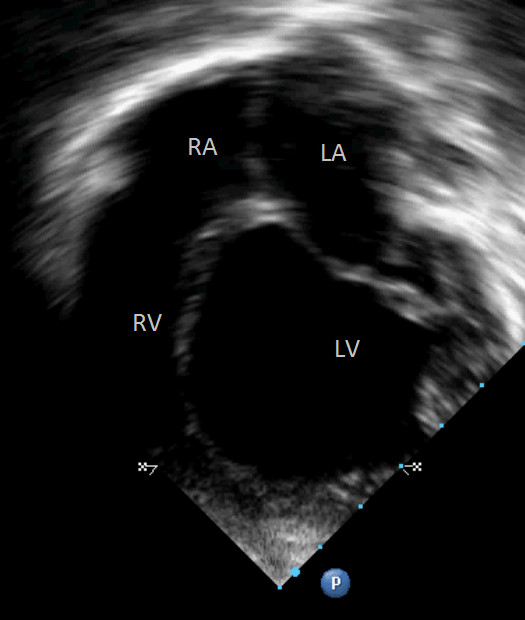
**Four-chamber echocardiographic image showing marked dilation of the left ventricle.** RA = right atrium, LA = left atrium, RV = right ventricle, LV = Left ventricle.

**Figure 6:**
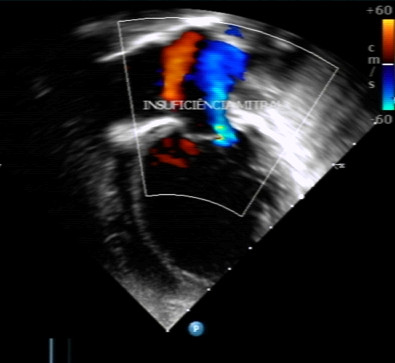
Echocardiographic image showing marked dilation of the left ventricle and mitral regurgitation (in blue) of moderate degree.

**Figure 7:**
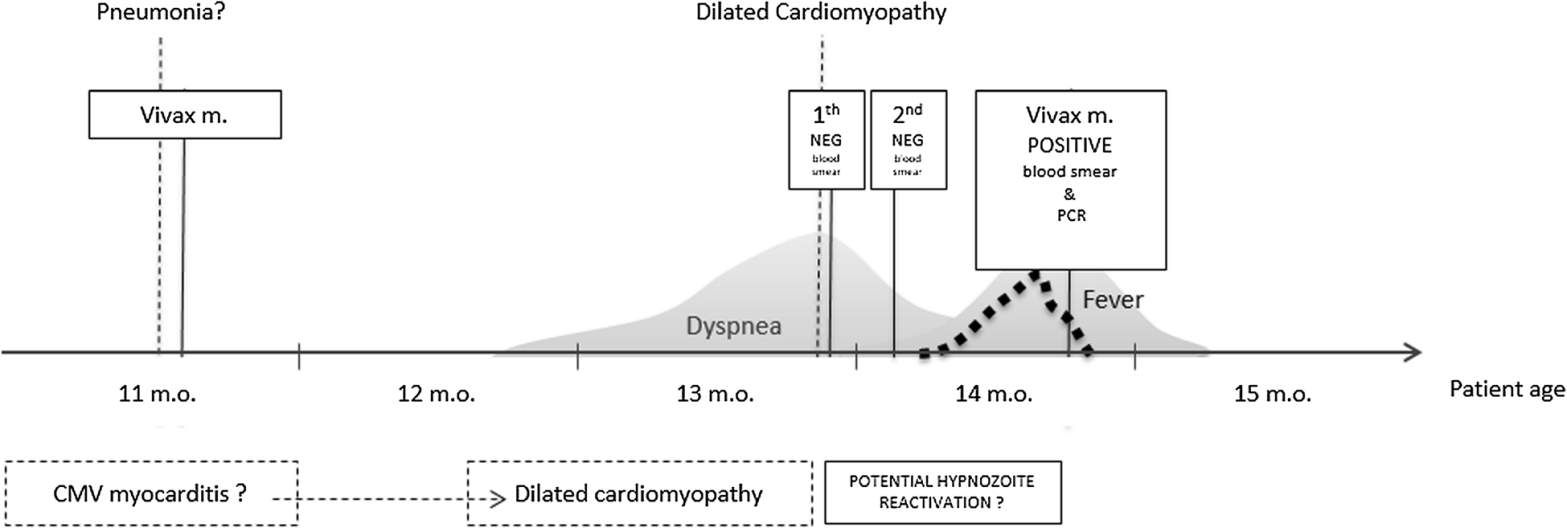
**Patient history with major clinical events, diagnosis and possible causal relationship.** The patient was admitted when she was 11 months old with respiratory symptoms and vivax malaria. She was treated and discharged home two days later without symptoms. After two months she started to have increasing dyspnoea and respiratory distress and was admitted again at 13 months old with a dilated cardiomyopathy and negative thick blood smears. During admission, there was an initial improvement of dyspnoea, but after a few weeks the dyspnoea worsened and she developed fever, with a positive smear to *P. vivax*. After malaria treatment with chloroquine and primaquine, there was important improvement of respiratory distress and fever. Positive serology for CMV suggest that she had a CMV-induced myocarditis around 11 months of age, progressing to symptomatic dilated cardiomyopathy, and later developing relapsing vivax malaria, with a severe clinical presentation.

Fever resolved on day 3 of chloroquine + primaquine treatment (October 6^th^) and dyspnoea improved from 7^th^ October onwards. There was a progressive significant clinical improvement with disappearance of respiratory distress between October 7^th^ and 14^th^. Despite this clinical improvement, cardiac echocardiogram remained mostly unchanged. A second echocardiogram performed on October 1^st^ showed dilated cardiomyopathy with severe systolic dysfunction, increased dilation of left atrium (31.9 cm), severe distension of left ventricle (48 cm × 39.5 cm), ejection fraction of 36% and severe mitral valve insufficiency. CMV IgM was 1.44 on October 25^th^. The third echocardiogram performed on November 1st showed severe dilation of left atrium (31 mm, Z score + 4.35), severe dilation of left ventricle (54 mm, Z score + 9.43), moderate mitral valve insufficiency secondary to ventricle dilation, severe systolic dysfunction with an ejection fraction of 41% and myocardial performance index of 1.03 (defined as the sum of isovolumetric contraction and relaxation time divided by the ejection time)(Figures 5 and 6). She continued to improve clinically and remained without clinical signs of respiratory distress despite maintaining cardiac dysfunction, and was kept on close treatment follow-up with prescription of furosemide, captopril, spironolactone, digoxin, and carvedilol. Figure 7 illustrates the most important events of the case.

## Discussion

The main features of this case are the dilated cardiomyopathy associated with respiratory distress and vivax malaria that presents later on the course of the disease. The cause of the dilated cardiomyopathy is problably CMV-induced myocarditis. Myocarditis can be acute, subacute or chronic, and it is characterized by a variety of symptoms, ranging from mild chest pain to cardiac shock and sudden death. In children, the most common symptoms are dyspnoea, vomiting, fever, and lethargy. Most of paediatric myocarditis is not recognized at the beginning, making the diagnosis even more difficult. Specific EKG change is increased PR interval, but ventricular and atrial overload are seen with the progression to dilated cardiomyopathy. In severe cases, cardiac insufficiency may develop because of acute severe injury or resulting dilated cardiomyopathy. In these cases, tachycardia, tachypnea and respiratory distress are frequent [19]. Although the gold standard examination for diagnosing myocarditis is myocardial biopsy, this procedure is not often performed. Ancillary diagnostic examinations include eletrocardiogram, echocardiogram and magnetic resonance of the heart [20]. Paediatric myocarditis are mostly caused by viral infections, namely enteroviruses (Coxsackie virus, echovirus and poliovirus). Other viruses are also associated with this lesion, such as parvovirus B 19, herpesvirus 6, influenza, para-influenza, adenovirus, *Epstein Barr* virus, and CMV. Less frequently, other non-viral agents, such as *Toxoplasma gondii* and *Trypanosoma cruzi,* can also cause acute myocarditis [21].

This child presented serial venous samples with increasing titres of seric IgM anti-CMV and high titres of IgG during admission. Studies on window interval with blood donors and blood recipients [22], and naturally infected immuno-competent patients [23] show that CMV genes are readily detectable while CMV-specific antibodies will take between three and five weeks after infection to be detected, supporting the hypothesis of CMV-related myocardial lesion in this child. Other studies show that IgM can take up to eight weeks to be detectable [24].

Several authors have described CMV-induced myocarditis. Evidence of CMV infection in ‘idiopathic’ dilated cardiomyopathy has also been shown [23,25]. A case of dilated cardiomyopathy in a two-month old infant with congenital CMV infection shows that progression from myocarditis to cardiomyopathy can occur in a few months [26], and that CMV specific IgM antibodies can be detectable at least 16 weeks after myocarditis has occurred [26].

It is possible that in this patient, CMV infection and acute myocarditis started in June, at the time of her first hospital admission, and that it was misdiagnosed as pneumonia because of respiratory symptoms. It has been shown that CMV can simulate pneumonia because of pronounced pulmonary congestion [25]. As pulmonary congestion and other symptoms of cardiac insufficiency increased and she progressed to dilated cardiomyopathy, the diagnosis of cardiac injury was made and she was referred to a larger hospital where other examinations and a proper diagnosis could be made.

The evolution of acute myocarditis to dilated cardiomyopathy has been explained by the occurrence of inflammatory lesion to the myocardial cell and adverse remodelling resulting in dilated cardiac chambers [27,28]. Experimental studies suggest that CMV infects the myocardial cell, which are then attacked by macrophages and natural killer cells, and later on by T lymphocytes, around 15 and 30 days post-infection [28,29]. After that, there is progression to fibrosis, cavity enlargement and heart failure, between 30 and 90 days post-infection [28], with persistence of viral genome in the heart even after 90 days of infection [28]. Sakaguchi *et al*. [26] described a case of congenital infection in which impaired cardiac function was detected at 30 weeks of gestation already. In the present case report, the time between the first hospital admission with fever and respiratory symptoms, and the respiratory distress leading to the second hospital admission was between four to eight weeks, supporting the hypothesis that this was a case of CMV-induced myocarditis progressing to dilated cardiomyopathy.

Besides having a pre-existing severe cardiac disease, this child also developed vivax malaria. Criteria for recognizing severe vivax malaria are now being discussed in the literature. Series of case reports and review of literature show a range of clinical and laboratory findings that are frequently seen in patients with severe vivax malaria [10,11,30]. These parameters included severe thrombocytopaenia [31,32], variable degrees of anaemia [5,33,34] (including severe anaemia with haemoglobin level <5 g/dL), lung injury with respiratory distress (mainly in children) [2,5,30,35,36], renal failure and jaundice [31,33,37], and less frequently, coma or cerebral malaria [2]. In this case, there was an acute drop in haemoglobin levels, together with worsening of respiratory symptoms. Although these changes do not meet the criteria currently used for severe malaria, the appearance of this second episode of vivax malaria contributed to worsening of clinical conditions.

In this case, the patient was already in respiratory distress, possibly as a result of CMV infection leading to dilated cardiomyopathy and pulmonary congestion, which worsened with the appearance of peripheral *Plasmodium* parasitaemia. Interestingly, treatment of malaria reduced the respiratory distress substantially, which confirms the role of *Plasmodium* in increasing the previous pulmonary injury she presented at admission. The mechanism of vivax-induced respiratory distress is currently attributed to cytoadherence to the lung microvasculature [38] and increased permeability of the alveolar-capillary membrane caused by sequestration and inflammatory response to the *Plasmodium* and its treatment [39], which is supported by *post-mortem* findings in Brazil [40]. On the other hand, malaria can also impairs cardiac function. Tachycardia, diminished ejection fraction and impaired left ventricular function were frequent in Kenyan children with severe falciparum malaria, and after treatment a significant improvement in cardiac function was observed. The aetiology of such cardiac depression is not clear, and may be related to cytokine release, acidosis, hypovolaemia or sequestration of red cells in myocardial vessels [41]. There are a few other reports of myocardial dysfunction caused primarily by malaria, all of them related to severe *Plasmodium falciparum* infection [42-44] and one caused by experimental malaria [45].

This one-year old child had a vivax malaria episode in June and another episode in October. Since she had a prolonged admission stay (23 days) with two negative thick blood smears and there is no malaria transmission in Rio Branco (where the hospital was located), it is highly possible that the patent parasitaemia detecting on 4 October, and the preceding seven days of fever, were the result of hypnozoite reactivation. Vivax malaria treatment was administered orally and it is possible that treatment with primaquine at home was not adequate in June, which would favour early relapse when facing a systemic disease [46]. It is less likely that recrudescence would occur, since chloroquine treatment was performed inside the hospital and most of recrudescence cases are seen within a month after incomplete treatment.There is scarce data on vivax relapses in the Brazilian Amazon. In 1991, Boulos *et al.*[47] estimated a 7% rate of relapsing vivax episodes, occurring up to 90 days after the first episode. There are no other recent reports for data comparison. Since albeit had not been possible to do rapid diagnostic tests or molecular testing for malaria before October 4^th^, it is conceivable that she had a very low *P. vivax* parasitaemia that was missed at hospital admission, but even in this situation, a relapsing malaria would be feasible due to improper treatment in June. In Brazil, access to anti-malarials is controlled by the Ministry of Health, and chloroquine usage is only allowed in two very defined situations: following a positive diagnostic test or as prophylaxis for recurrent vivax malaria after the second episode when primaquine cannot be used (children under 6 months of age and pregnant women) [16].

It is described that repeated episodes of malaria can cause the removal of both infected and uninfected red blood cells, which could explain the anaemia observed at the second hospital admission, together with some degree of iron deficiency caused by low intake. The substantial drop of haemoglobin levels around October 4^th^, on the other hand, is directly related to the severity of vivax malaria [30], and can be caused by haemolysis, impaired haemopoiesis or spleen sequestration. This child also presented lymphopaenia, detected in October 3^rd^, one day before she had a positive thick smear form malaria. Although it is not a correlate of severe disease, lymphopaenia is frequent in malaria patients [48].

The aetiology of diarrhoea was not formally investigated, but it is possible that she had an antibiotics-associated colitis, or even *Clostridium difficile*-associated diarrhoea. *Clostridium difficile* diarrhoea is the most common cause of diarrhoea associated with antibiotic usage, and the most common symptoms in children are liquid stools that resolve in 30 days, while adults tend to have pseudomembranous colitis much more frequently [49]. This child was started empirically on vancomycin and diarrhoea resolved three days later, while fever continued to exist. Changes in haemogram from September 23^rd^ showed increased leucocyte count with increased segmented neutrophils, supporting the hypothesis of an antibiotic-associated *C. difficile* diarrhoea [49].

Technical limitations that are common in the Amazonian health care system prevented a more detailed investigation of the case. Malaria smears should have been performed earlier and more often during the course of admission in this case, and maybe this would have prevented the acute drops in haemoglobin levels and platelet numbers, and the worsening of cardiorespiratory function. The aetiology of diarrhoea was not properly investigated either. Social conditions will make the follow-up of this child with cardiac insufficiency more difficult, since at her home area there is no cardiologic health care available. It is worth pointing out that in malaria-endemic settings, socio-economic inequalities can facilitate the occurrence of co-morbidities and at the same time may hinder proper health care.

## Conclusion

This case portrays the concomitance of two important infectious diseases in a young child. While CMV infection is a cosmopolitan infectious agent, vivax malaria is a disease associated with social inequities, and should be investigated in all patients with similar settings. Health care provided to this patient at the beginning of clinical course was limited because of inherent health care access in the Amazon, but despite that, medical efforts were able to keep her health condition stable.

## Consent

Written informed consent was obtained from the parents of the patient for publication of this case report and any accompanying images. A copy of the written consent is available for review by the Editor-in-Chief of this journal.

## Competing interests

The authors declare they have no competing interests.

## Authors’ contributions

ACM, JBL, LMNS, and TCM provided clinical care to the patient and pursued the initial clinical investigation, interpretation of exams and malaria diagnosis. LNF and RSM carried out the molecular diagnosis of *Plasmodium* infection. MCVR and RBR performed the cardiological evaluation and diagnosis and provided final health care to the patient. MdaSN participated as an infectious disease consultant expert. ACM and MdaSN conceived the case report, participated in its design and coordination and wrote the manuscript. MCVR and RBR helped to draft the manuscript. JBL, LMNS, TCM, LNF, and RSM revised the manuscript critically for important intellectual and clinical content. All authors approved the final manuscript.
